# TE Hub: A community-oriented space for sharing and connecting tools, data, resources, and methods for transposable element annotation

**DOI:** 10.1186/s13100-021-00244-0

**Published:** 2021-06-21

**Authors:** The TE Hub Consortium, Tyler A. Elliott, Tony Heitkam, Robert Hubley, Hadi Quesneville, Alexander Suh, Travis J. Wheeler

**Affiliations:** 1 Centre for Biodiversity Genomics, University of Guelph, Guelph, ON Canada; 2 Faculty of Biology, Technische Universität Dresden, 01069 Dresden, Germany; 3 Institute for Systems Biology, Seattle, WA USA; 4 Université Paris-Saclay, INRAE, URGI, 78026 Versailles, France; 5 School of Biological Sciences, University of East Anglia, Norwich, UK; 6 Department of Organismal Biology, Uppsala University, Uppsala, Sweden; 7 Department of Computer Science, University of Montana, Missoula, MT USA

**Keywords:** Transposable elements, Classification, Annotation, Database, Software, Collaboration, Community

## Abstract

Transposable elements (TEs) play powerful and varied evolutionary and functional roles, and are widespread in most eukaryotic genomes. Research into their unique biology has driven the creation of a large collection of databases, software, classification systems, and annotation guidelines. The diversity of available TE-related methods and resources raises compatibility concerns and can be overwhelming to researchers and communicators seeking straightforward guidance or materials. To address these challenges, we have initiated a new resource, TE Hub, that provides a space where members of the TE community can collaborate to document and create resources and methods. The space consists of (1) a website organized with an open wiki framework, https://tehub.org, (2) a conversation framework via a Twitter account and a Slack channel, and (3) bi-monthly Hub Update video chats on the platform’s development. In addition to serving as a centralized repository and communication platform, TE Hub lays the foundation for improved integration, standardization, and effectiveness of diverse tools and protocols. We invite the TE community, both novices and experts in TE identification and analysis, to join us in expanding our community-oriented resource.

## Introduction

Transposable elements (TEs) are mobile and often-replicating genetic elements that make up a significant fraction of most eukaryotic genomes (for reviews, see [1, 2]). Their study is important in genome research [3] as they can be viewed as motors of evolution [4], regulators of gene control [5], and as genomic building blocks [6]. Over the years, the field has accumulated a plethora of databases, software, classification systems, and annotation guidelines [7, 8]. These options provide researchers with the tools to discover and investigate TEs in existing and new genome sequences, and to update and revisit already characterized TEs. However, in-depth TE detection and analysis is laborious, and largely requires significant expertise in TE biology.

The expansive and diverse collection of tools and methods often leads to at least two significant problems for even the most experienced bioinformatician. First, the set of available choices can be overwhelming, leaving the researcher unsure of preferred methods for analyzing particular data types. Second, the multitude of databases and tools often suffers from compatibility concerns in both nomenclature and output format [9, 10]; this is especially true for databases focusing on different TE types or host organisms.

Yearly conferences and workshops [11–13] provide some relief from these pressures – tool/database developers can meet and find common ground, while users of these resources can gain valuable exposure to new methods and best practices. Even so, the transient and punctuated nature of these meetings, combined with rapid developments in the TE field, leave much to be desired in terms of collaboration, interactivity, and persistent documentation of existing methods and best practices.

**Fig. 1:**
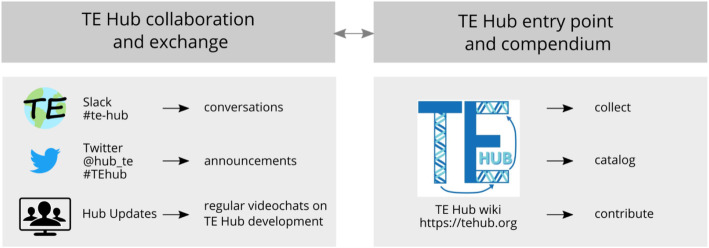
TE Hub’s core components help to establish an open and collaborative platform for documenting and discussing TE-related methods

We have initiated TE Hub as an answer to these challenges. TE Hub is envisioned as a community-oriented framework that will serve as a resource for novice and expert TE researchers. For novices, TE Hub gives practical insight into available TE resources and methods, and for experts and developers, it provides a platform for increased communication and improved integration of methods and databases (see Fig. 1). Specifically, TE Hub is designed to support the TE community in three ways:

1. We have developed a website (https://tehub.org) that serves as an up-to-date compendium of information about TE research; the site is managed by an open wiki framework, so that all members of the TE community can contribute in an open, nimble, and transparent manner.
2. We have established a framework for focused communication among and with TE Hub contributors, via a messaging channel (#te-hub) housed in the larger TransposonsWorldwide Slack workspace [14], and a dedicated Twitter account (@hub_te).
3. The website, supplemented by open bi-monthly meetings, lays the foundation for development of a federated mechanism for integrating tools, databases, and resources in a way that will, over the long term, improve and standardize their value to the TE research community.

In the following sections, we provide further details about these components of TE Hub, describing the current state and establishing a vision for its future. TE Hub is a community-oriented resource, and we wrap up by describing how interested TE experts and novices can get involved.

## The TE Hub website

The focal point of TE Hub is the website: https://tehub.org, which is intended to serve as a compendium of tools, databases, and other features of value to TE researchers, both novice and expert. The site content is managed via a wiki system, so that researchers can contribute to the content in an open, timely, and transparent fashion. TE Hub data is roughly organized along the following facets of TE-related information:

1. Classification. This section captures a collection of established classification schemes, both overarching and specific for certain hosts and TE-types. At the time of this writing, the five most commonly used overarching TE classification systems [15–19] are represented, along with four specialized classification systems [20–23]. Furthermore, a collection of 519 TE lineages is captured, each with at least one relevant reference in the literature. These will be particularly useful to TE novices, aiming to understand common nomenclature and the relationships between alternative systematic hierarchies.
2. Databases. This section compiles a list of databases for the storage of sequences and metadata associated with TEs, with links to each database and corresponding publication, along with a description of the represented repeat types and taxonomic groups. At the time of this writing, 150 databases are represented.
3. Tools. This section compiles a list of software for the detection, annotation, analysis, simulation, and visualization of TEs. Websites, preprints, and journal articles are linked, and associated with keywords. At the time of this writing, 505 tools are represented.
4. Protocols. Over time, this section will hold a collection of suggested protocols for use by researchers engaged in TE identification and annotation. The lack of carefully-crafted, discoverable, open-access protocols is an impediment to novice TE annotators. At the time of this writing, two protocols are listed; we expect this section to be substantially expanded in the coming months, and invite experienced annotators to contribute their mature and open access protocols.
5. Journals and Conferences. These sections capture a collection of journals that often publish TE-relevant articles, and a (community-maintained) listing of upcoming TE-related conferences.
6. Outreach and Teaching Resources. These sections hold a collection of educational resources that are intended to provide background on TEs, course materials for TE-related classes and workshops, and links to public talks on TEs intended for a general audience.

Contribution to the TE Hub is strongly encouraged and requires ORCID authentication. Dependency on ORCID ensures that content can be credited to each contributor, and represents a small barrier to contribution, as creation of an ORCID account takes only a few minutes. All TE Hub content is made available under the CC-BY license (https://creativecommons.org/licenses/by/4.0).

## TE Hub communication channels

As a complement to the frequently updated but relatively static content of the TE Hub website, we have established mechanisms for scheduled and ad hoc communication about TE annotation resources and methods. These include:

1. The #te-hub channel, housed in the broader TransposonsWorldwide Slack workspace (https://transposonsworldwide.slack.com; currently with over 500 members). The #te-hub messaging channel is focused on the databases, software, and annotation methods central to TE Hub, leaving broader matters of TE biology to other TransposonsWorldwide channels. To insure against a records loss, conversations on the #te-hub channel will be regularly archived.
2. The @hub_te Twitter account (https://twitter.com/hub_te) will be used for TE Hub announcements, and the #TEhub hashtag will be adopted as a mechanism for highlighting Hub-relevant tweets.
3. ‘Hub Updates’ are video calls that serve as a regular medium for communication among database/methods developers and users of these methods. Meetings run for one hour, are held on a bi-monthly basis (organized transparently via the above Slack channel and Twitter account), and are open to all. These meetings have been ongoing since June 2020.

## A foundation for the future of TE annotation

Creation of the TE Hub wiki resource and communication channels are the first step in a larger plan to develop a framework for improved integration of disparate TE datasets, tools, and resources. TE Hub is not, and is not intended to become, a replacement for individual TE databases (e.g. Repbase Update [15], DFAM [18], RepetDB [24], GyDB [20]) or annotation methods (e.g. RepeatModeler2 [25], REPET [26], RepeatExplorer2 [27]). Rather, the vision is that these first TE Hub developments will lay the foundation for future efforts to build a common language around diverse databases, establish a system for improving interoperability of independent TE identification and annotation software that capitalizes on each tool’s individual strengths, and develop an increasingly robust catalog of annotation protocols, all with the goal of improving the ease and effectiveness of annotation for a maximally-broad diversity of organisms. In the meantime, the current compendium of methods and data will serve as a bridge to the future for TE annotators.

## Call for engagement and contribution

TE Hub has grown out of a grassroots effort to expand international collaboration in the development of TE identification and annotation methods, to broaden and unify their applicability to non-model organisms, and to establish a comprehensive catalog of TE resources that can be easily updated by members of the community. The regular Hub Update meetings grew out of discussions in the Slack channel, and led to the content and vision described here. While this effort has been driven by a small steering committee rising out of these Hub Update meetings, the future of TE Hub depends on engagement and contribution from others in the community.

We invite the TE community, both novice and expert TE researchers, to join us in expanding our community-oriented resource. Please follow us on Twitter: @hub_te (https://twitter.com/hub_te), and visit https://tehub.org/volunteer for more information about contributing to the future of TE Hub. To fully engage, two registrations are recommended:

1. Join the TransposonsWorldwide Slack workspace (https://transposonsworldwide.slack.com) and find the #te-hub channel under “Browse channels”. This will allow you to track and contribute to ongoing conversations related to TE Hub content development, and to receive notification of upcoming ‘Hub Update’ discussions and notes.
2. Register on the TE Hub wiki, using your ORCID iD (https://orcid.org/). Though this is not required in order to view TE Hub content, it will enable your future contribution of content by editing appropriate individual wiki pages.

## Data Availability

Not applicable.
